# Ambard’s Constant

**Published:** 1914-05

**Authors:** 


AMBARD'S CONSTANT. George Hermyle Bril, *Le Jour. de Med. et de Chir.*, de Montreal, November, 1913. Urea is no longer regarded as a product of bodily metabolism but as corresponding to the N of the diet, so that, indeed, the diet can be approximately checked by the urea estimations. Amard's constant represent the relation of the urine to the blood, in urea, at a given period. The full method is quite complicated so that we attempt only to abstract the principal points. By catheter, the urine is collected for a period of exactly ten minutes and its urea is compared with that of the blood, drawn immediately afterward. There is some difference of opinion as to whether the blood should be drawn from a vein or by wet cupping. The author considers the latter method more representative of the average blood, the urea content of the arterial blood being approximately identical whereas the venous blood differs in urea content and that of extractives in different parts and under different conditions, as of exercise.

Ambard's Constant is the per mille of urea in the blood divided by the square root of the following product: the total 24-hour output, or rather the estimated 24-hour output at the rate observed in the experiment, times 70 kilograms divided by the actual weight of the patient, times 1/5 the square root of the per mille content of the urine in urea.

